# Causes of accelerated High-Tide Flooding in the U.S. since 1950

**DOI:** 10.1038/s41612-023-00538-5

**Published:** 2023-12-15

**Authors:** Qiang Sun, Sönke Dangendorf, Thomas Wahl, Philip R. Thompson

**Affiliations:** 1 Department of River-Coastal Science and Engineering, Tulane University, 627 Lindy Claiborne Boggs Center, 6823 St. Charles Avenue, New Orleans, LA 70118 USA; 2 Department of Civil, Environmental and Construction Engineering and National Center for Integrated Coastal Research, University of Central Florida, Orlando, FL USA; 3 Department of Oceanography, University of Hawai’i, Honolulu, HI USA

**Keywords:** Climate-change impacts, Environmental impact

## Abstract

The U.S. coastlines have experienced rapid increases in occurrences of High Tide Flooding (HTF) during recent decades. While it is generally accepted that relative mean sea level (RMSL) rise is the dominant cause for this, an attribution to individual components is still lacking. Here, we use local sea-level budgets to attribute past changes in HTF days to RMSL and its individual contributions. We find that while RMSL rise generally explains > 84% of long-term increases in HTF days locally, spatial patterns in HTF changes also depend on differences in flooding thresholds and water level characteristics. Vertical land motion dominates long-term increases in HTF, particularly in the northeast, while sterodynamic sea level (SDSL) is most important elsewhere and on shorter temporal scales. We also show that the recent SDSL acceleration in the Gulf of Mexico has led to an increase of 220% in the frequency of HTF events over the last decade.

## Introduction

The U.S. coasts have experienced rapid increases in flooding days during recent decades^1–7^. Under present mean sea level, at many locations, the combination of a regular spring tide and moderate onshore winds produces flooding of low-lying coastal zones^7–9^. This coastal flooding, also commonly referred to as High-Tide Flooding (HTF), disrupts people’s daily lives by blocking roads, forcing businesses to close temporarily, or damaging property. Though the impacts of an individual event may be minor, the costs aggregated over time can exceed those of less frequent extreme events at some locations^10^. Consequently, stakeholders and policymakers are concerned about the growing impacts of HTF, increasing the need for improved process understanding and projections^11^ that enable decision-makers to evaluate the merits of coastal management strategies under various mitigation and adaption scenarios.

HTF occurs due to the combination of a high (spring) tide, (storm) surge, and relative mean sea level (RMSL) anomalies. Decadal periods of enhanced/reduced HTF have previously been connected to perigean and nodal modulations of tidal amplitudes^11^, tropic tides related to lunar and solar declination^12–14^, and interannual RMSL variability^4,11^. Particularly within bays and estuaries, anthropogenic alterations of tides have also contributed to sustained changes in HTF^15,16^. However, the increases in annual HTF days have widely been associated with RMSL rise increasing the baseline upon which tides and surges occur^14,17^ and reducing the gap to local flood thresholds^2^. Global mean sea level has risen by about 1.5 mm per year since 1900 due to (thermo-) steric expansion of the ocean and barystatic mass changes due to melting ice sheets and glaciers^18^; the rate of rise has further been accelerating since the 1960s^19–21^. Regionally and locally, there are significant deviations from the global mean due to processes such as (i) variations in Gravity, Rotation, and Deformation (GRD) that accompany contemporary barystatic mass changes (due to ice mass loss, or changes in terrestrial water storage) and cause instantaneous changes in the geoid and the solid Earth (see ref. ^22^ for further details), (ii) sterodynamic variations due to changes in ocean circulation, atmospheric pressure and wind forcing, and (iii) Vertical Land Motion (VLM). Along U.S. coasts, these processes contributed to RMSL rise ranging from 0.9 to 7.0 mm per year since 1950^4,23–25^, providing a plausible explanation for the rapid increases in HTF^7^. Although previous studies illustrate a close relationship between RMSL rise and the increases in HTF days, the relative contributions of regional and local processes are not yet fully understood.

Knowledge about the contributors to changes in HTF is important for multiple reasons. Not all of the observed RMSL changes are due to anthropogenic climate change; for instance, VLM occurs due to both human actions—such as withdrawals of groundwater, oil, or gas that destabilize the ground^26^—but also natural processes such as Glacial Isostatic Adjustment (GIA, the ongoing adjustment to the melting of large ice sheets after the last glacial ice age). While anthropogenic VLM can be mitigated locally (for instance, by avoiding further fluid withdrawals), natural VLM will persist into the foreseeable future. Thus, even with the strongest climate mitigation efforts, HTF will further increase in many places and therefore requires adaptation efforts. In addition, better understanding of the contributions of various processes to HTF at different timescales allows for improved physics-based short-term forecasts and/or long-term projections of impacts.

## Results

### HTF and the role of RMSL changes

**Fig. 1:**
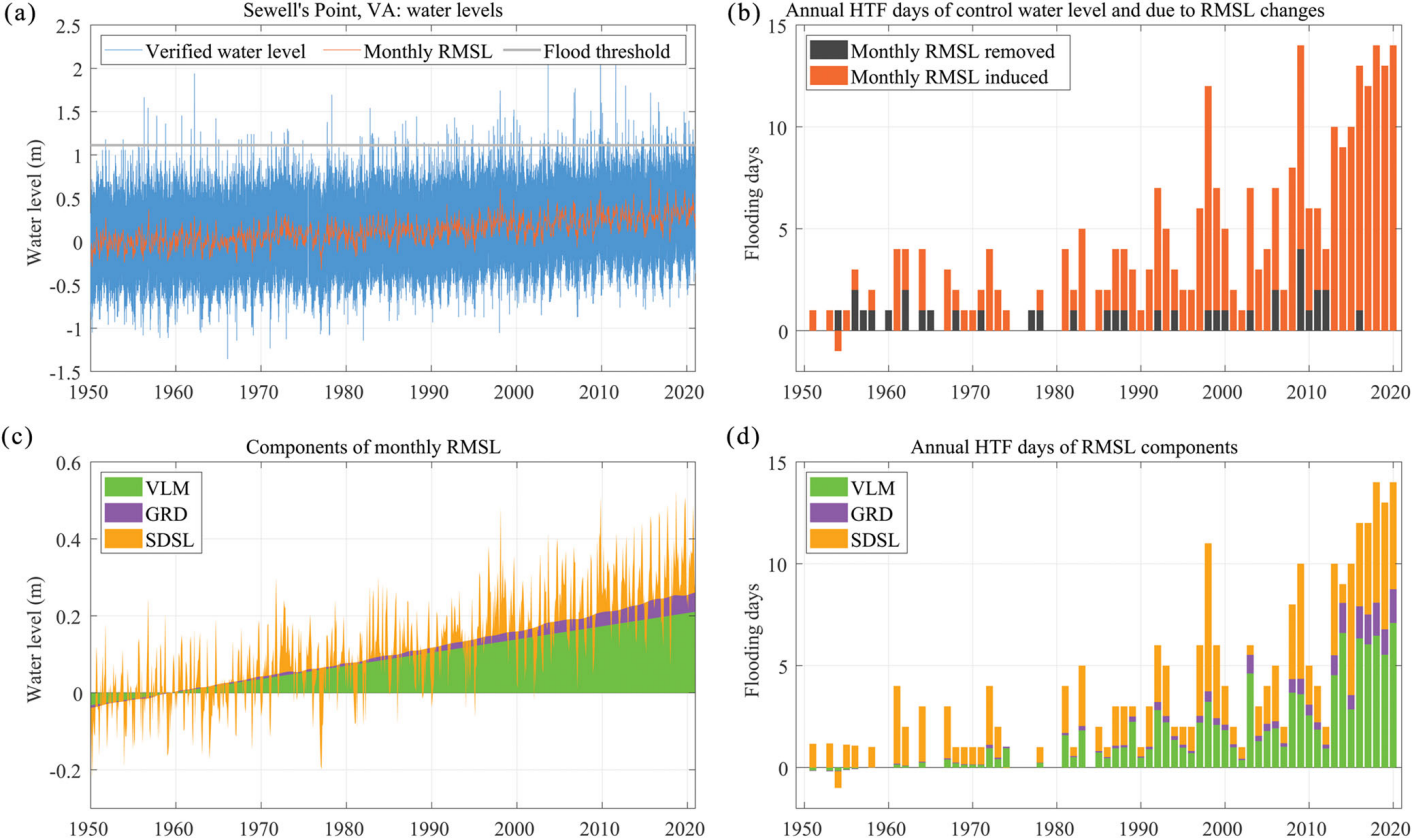
Water levels and annual HTF days at Sewell’s Point in Norfolk, Virginia. **a** Represents the verified water levels in blue, monthly RMSL in red, and flood threshold in gray. All water levels are referenced to the control period of 1950–1968. **b** Shows the annual HTF days for the water levels without monthly RMSL in black and the HTF days induced by monthly RMSL changes in red. The summation of the stacked bars in each year equals the detected annual HTF days. **c** Indicates the individual processes to the local RSML (i.e., the top edge of the green patch is VLM, the top edge of the purple patch is VLM + GRD, and the top edge of the orange patch is SDSL + GRD + VLM). **d** Reveals the annual HTF days for each process. The summation of the HTF days of all processes in each year equals the RMSL-induced HTF days in **b**.

Here, we assess changes in HTF from 1950 to 2020 along the coasts of the contiguous United States (CONUS) and six Pacific islands, including Hawaii, with a particular focus on the role of RMSL and its individual contributing processes using minor flooding thresholds from the NOAA National Ocean Service (NOS)^7^ (Supplementary Fig. 1, see Methods for further details on data selection criteria). Thresholds also exist from the National Weather Service (NWS). NWS thresholds are tied to local impacts (using expert knowledge), but they are not available everywhere, and NOS thresholds have been suggested to be more suitable for studying broader vulnerabilities of HTF (results for NWS thresholds can be found in the Supplementary Fig. 2). The approach is illustrated at the Sewell’s Point tide gauge in Norfolk, Virginia (Fig. 1). To quantify the contribution of RMSL changes—including both long-term rise and seasonal to interannual fluctuations—to HTF, we calculate the annual HTF days with and without monthly RMSL referenced to 1950-1968 (hereafter; control period, see Methods). At Sewell’s Point, a total of 280 HTF days were detected, with a maximum annual count of 14 (Fig. 1b). But without RMSL changes, only 36 HTF days would have occurred, with a maximum annual count of four. Thus, while the combination of high tides and surges without RMSL would still have led to HTF, the significant reduction in HTF days confirms that the vast majority of HTF events since 1950-1968 are linked to RMSL changes at Sewell’s Point.

**Fig. 2:**
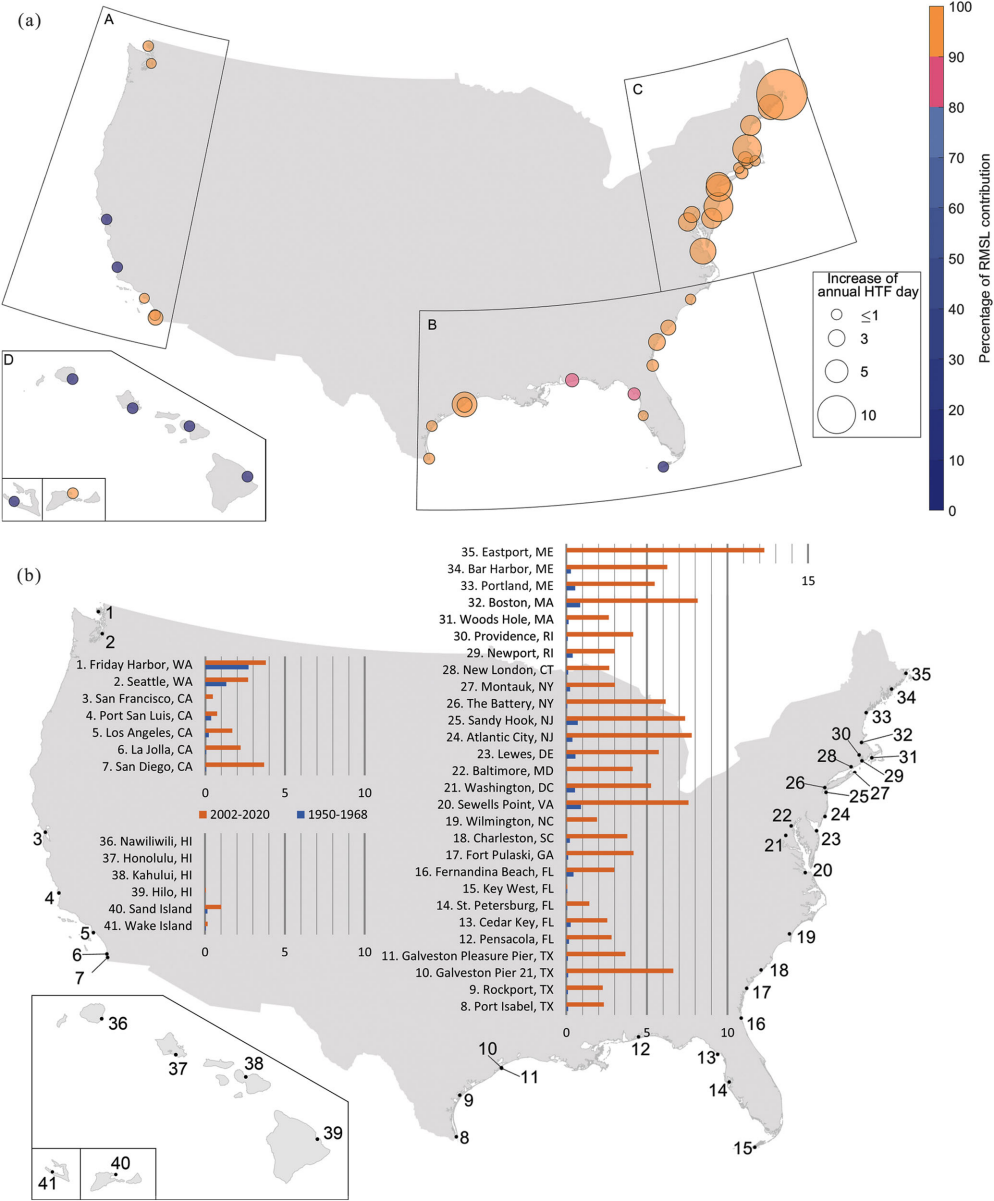
Increases of annual HTF days and RMSL contributions. **a** The radius of markers along the coasts shows increases in annual HTF days from 1950–1968 to 2002–2020, resulting from the verified water levels and averaged over the two 19-year periods. The colors of the markers represent the percentage contributions of RMSL changes to the increases in the HTF days. **b** Annual HTF days due to RMSL changes averaged for the control period (1950–1968) in blue and for the recent period (2002–2020) in orange. The selected sites are numbered along the coasts of the CONUS and Pacific islands. The stations with less than one day of increase in HTF in **b** only show minimized marks in **a**.

To get a more comprehensive picture of HTF along the coasts of the CONUS and Pacific islands, we first show the difference in annual HTF days averaged for two 19-year periods: 2002-2020 and the control period (Fig. 2a). Compared to the control period, the annual counts of HTF days increased by more than 2.5 days at 20 out of 41 sites. The largest changes in HTF occurred along the U.S. east coast, with a cluster of particularly large increases in the Mid-Atlantic Bight and the Gulf of Maine, which is consistent with earlier works^27^. In Atlantic City and Boston, for instance, the average HTF occurrence has increased by eight days per year compared to the control period. In Eastport, the recent annual count of HTF is 13 days more than in the control period. Along the U.S. Southeast and Gulf Coasts, the median of increases in the annual HTF is generally smaller with changes ranging from 1.3 days (St. Petersburg, Florida) to 6.5 days (Galveston Pier 21, Texas). A similar picture is available along the CONUS west coast with moderate changes ranging from 0.3 days in Port San Luis, California, to 3.6 days in San Diego, California.

**Fig. 3:**
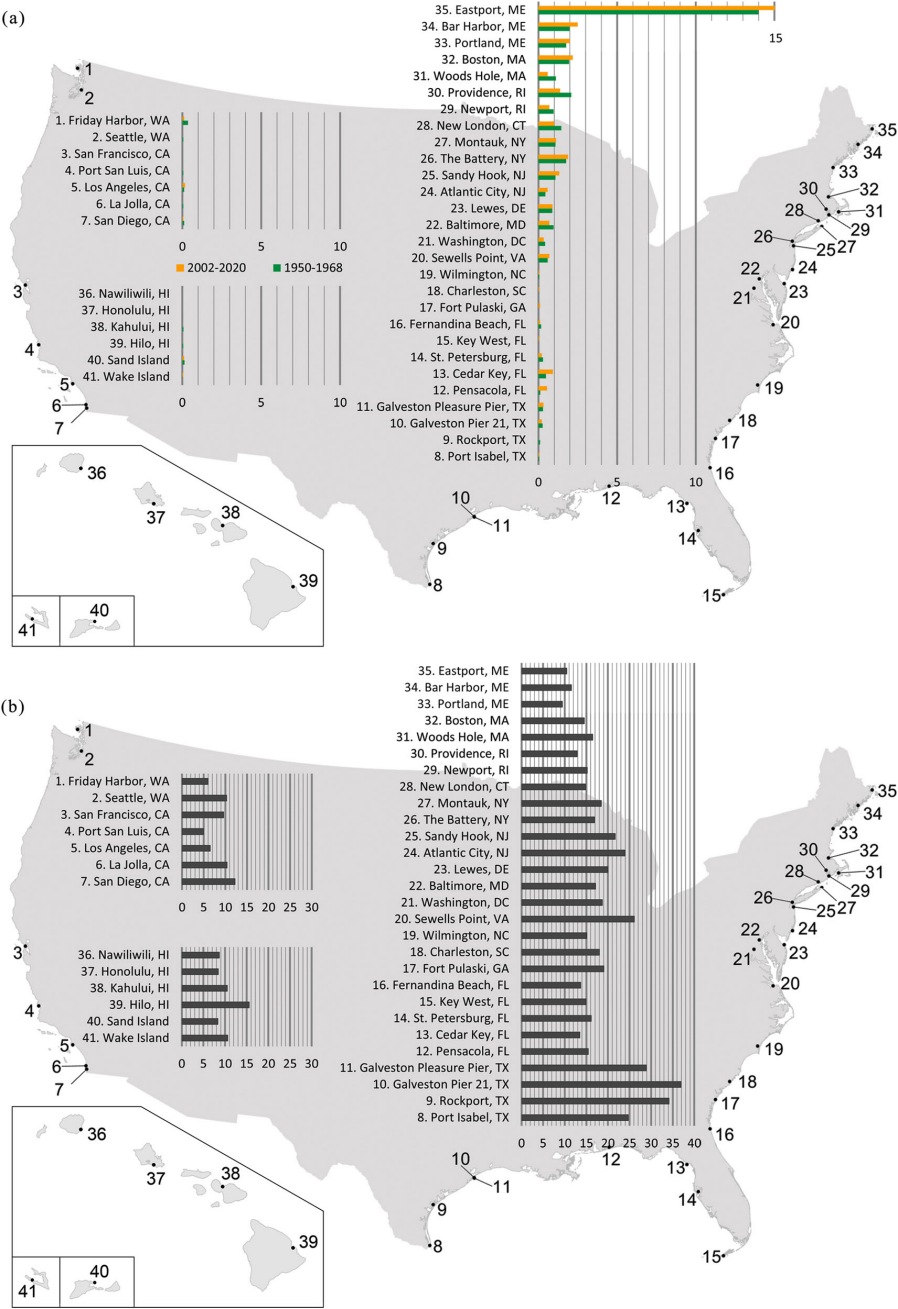
The average annual HTF days resulting from the water levels excluding RMSL changes, and RMSL rise in the units of centimeters. **a** Similar to Fig. 2b but representing the HTF days detected with control water levels (monthly RMSL removed) for 1950–1968 in green and 2002–2020 in yellow. **b** The increases in the RMSL are calculated as differences in the mean water levels of two 19-year periods: 1950–1968 and 2002–2020.

**Fig. 4:**
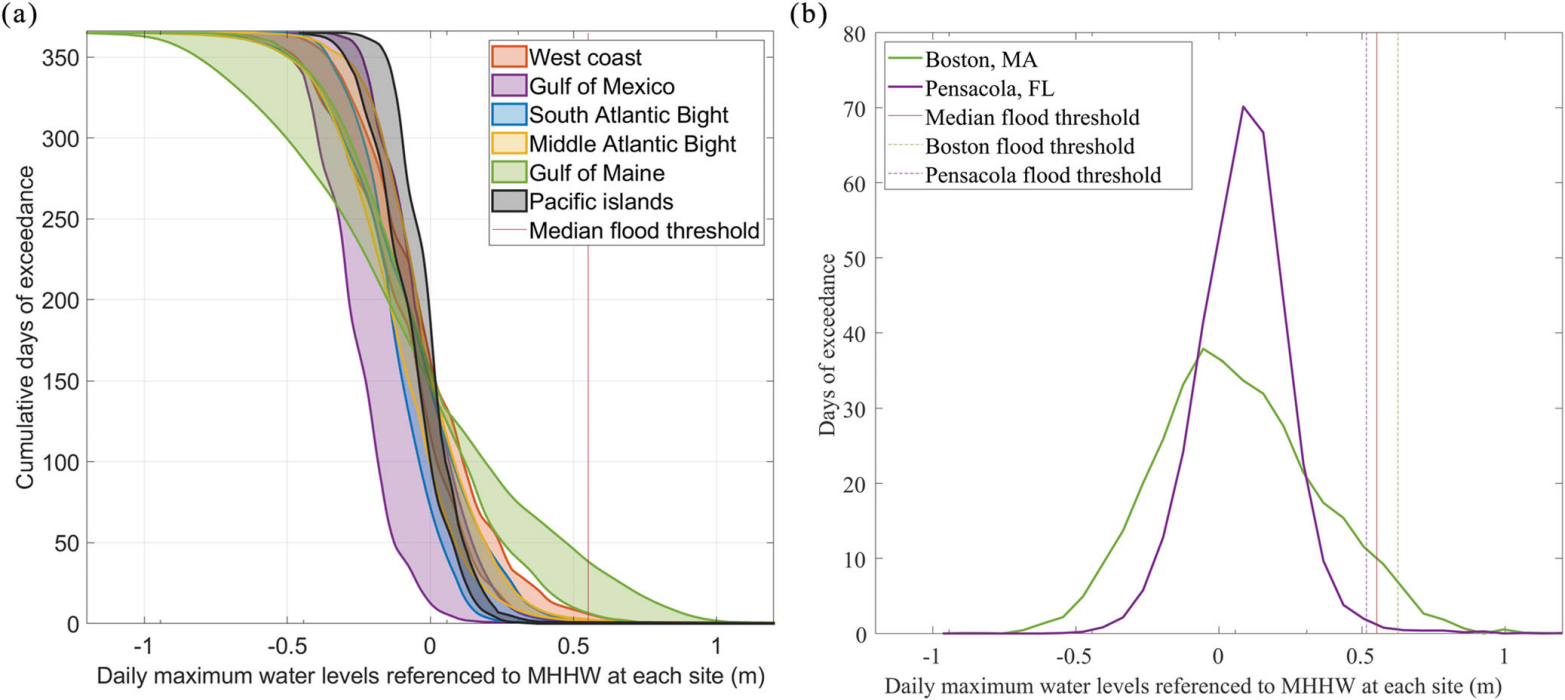
Exceedance of daily maximum water levels referenced to MHHW. **a** The colored patches represent the cumulative exceeding days of daily maximum water levels in the control period (1950–1968) in different coastal regions of the CONUS and Pacific islands. The horizontal axis indicates the daily maximum water levels above the MHHW of each site. **b** Distribution of the daily maximal water levels and flood thresholds in Boston, Massachusetts, and Pensacola, Florida. The format of horizontal axis is the same as in plot **a**.

Next, we isolate the contribution of RMSL changes to the increases in HTF days (Fig. 2b). Without RMSL changes, the HTF occurrences are not remarkably different (with ±1 day between the two periods, Fig. 3a and Supplementary Table. 1). This indicates that the majority of the long-term increases in the frequency of HTF days is attributable to RMSL changes (even though we note that individual events are dominated by the contributions of tides and surges^15^). The additional comparisons between linear trends of RMSL and 95th percentile of tides (e.g., due to observed changes in tidal range^28,29^) and non-tidal residuals further indicate that the RMSL rise is the dominant long-term driver increasing the coastal sea level, which leads to growth of the HTF days (Supplementary Fig. 5). Indeed, RMSL changes are responsible for over 84% of the HTF increases at 33 out of 41 sites (Fig. 2a). The eight remaining sites did not experience any notable changes over the investigation period (Supplementary Table 1). Interestingly, the spatial pattern of RMSL rise (Fig. 3b) only partially reflects the spatial pattern of increases in the HTF days with a moderate correlation of r = 0.57. For instance, some of the largest increases in HTF days are detected in the Mid-Atlantic Bight, despite that sites in the Gulf of Mexico have experienced the most substantial RMSL rise (2–3 times higher depending on location). Similarly, while the Gulf of Maine has experienced less RMSL rise, some sites show even higher increases in HTF occurrences than in the Mid-Atlantic Bight. There are two possible reasons for the moderate spatial correlation. First, the NOS flooding thresholds, which are designed to reflect the broader vulnerability of U.S. coasts to HTF^7^, depend on the tidal great diurnal range^4^. The latter varies between locations leading to different responses in regional HTF occurrence. However, using the more localized thresholds from the NOAA National Weather Service (NWS) (Supplementary Fig. 1) does not improve the spatial correlation (r = 0.4), suggesting that the flooding threshold choice is not the main reason. Second, the variances of the daily maximum water levels are notably different between locations due to the varying characteristics of tides and non-tidal residuals at each site (due to different wind and pressure forcing, and varying shelf widths)^4^, leading to varying shapes of the water level distributions. To illustrate this influence, we show distributions of daily maximum water levels for different sub-regions (Fig. 4a). A slowly-decaying distribution, as found in the Gulf of Maine, indicates large variances in the daily maximum water levels, while a fast-decaying distribution, as found in the Gulf of Mexico, reveals small variances in the water levels (Fig. 4b). The differences in the water-level variances result in distinct regional responses in HTF occurrences to RMSL rise. With small water-level variances, the Gulf of Mexico needs substantial RMSL rise to create rapid increases in the HTF days from the control period. In contrast, with large water-level variances, the Gulf of Maine has already seen sizeable HTF days in the control period, and comparably little RMSL increases have resulted in more rapid growth in HTF (Fig. 4a). The differences in regional water-level variances also have an important implication for future projections of HTF days. While the regions with small water-level variances (most notably in regions with diurnal and mixed tides, such as the Gulf of Mexico and the U.S. west coast) had previously experienced slower increases in the number of HTF days, they will grow more rapidly once the RMSL rise surpasses a certain threshold. This is in line with the results of ref. ^11^, who showed that the Gulf of Mexico will reach a tipping point of rapid increase in HTF earlier than other regions. Therefore, the amount of RMSL rise and the regional water-level variances both matter for the past increases in HTF days and their future projections.

**Fig. 5:**
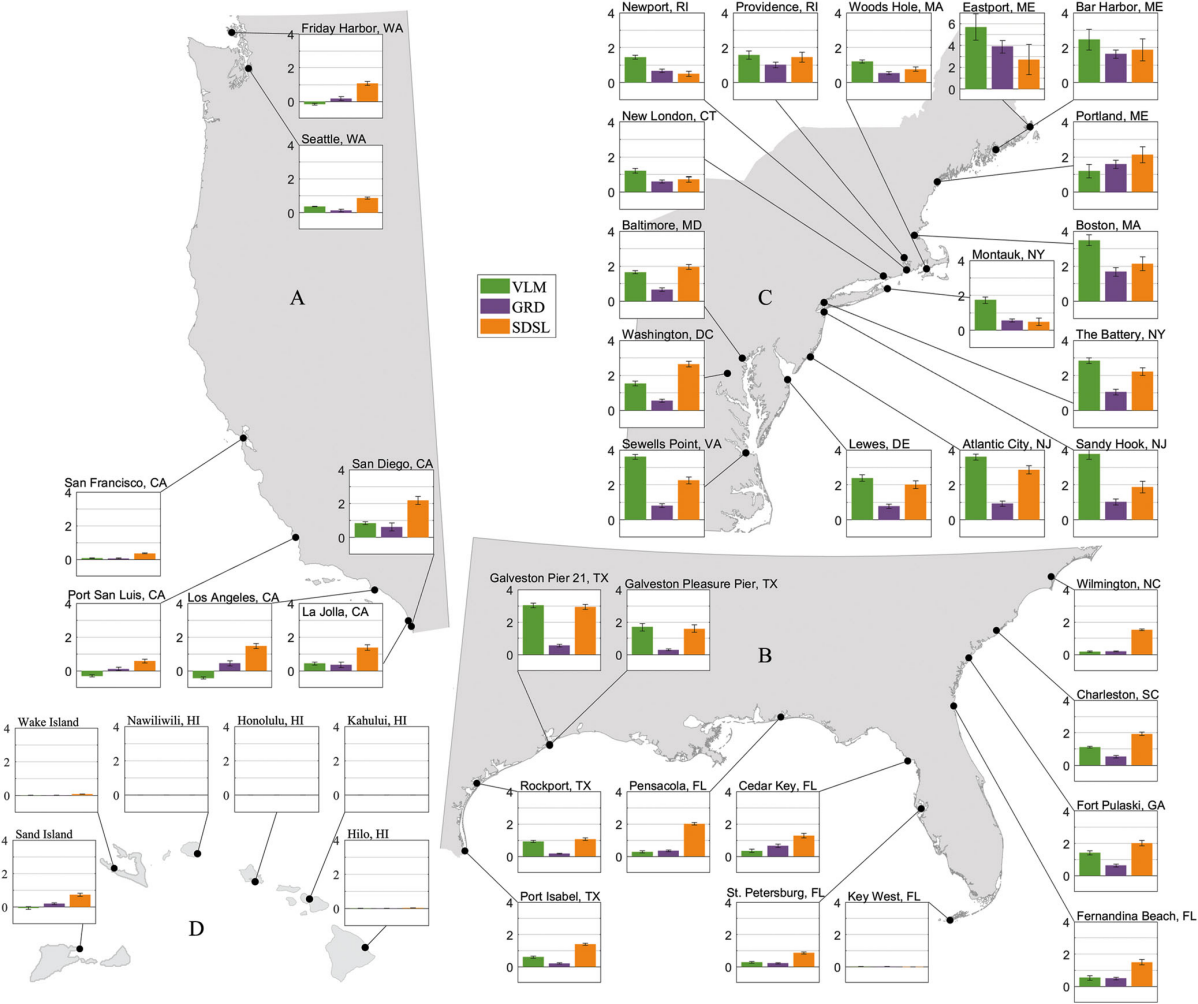
The annual HTF days of individual processes. The bar plots in each subpanel reveal the mean annual HTF days increase resulting from each process in RMSL changes averaged over two 19-year periods from 1950–1968 to 2002–2020. The error bars represent the one standard deviation of the HTF days. For better visibility, plots zoom in for four regions, A, B, C, and D, indicated in Fig. 2a.

### Process-based HTF decomposition

Having identified RMSL changes as the main contributing factor to increases in HTF days along the U.S. coasts, we next assess the contributions by individual budget components. We decompose RMSL into three components: GRD (i.e., the fingerprints of barystatic RMSL change^30^), sterodynamic sea level (SDSL), and VLM. We assume that their relative importance can be linearly transferred to HTF days (see Methods). Two components, GRD and VLM, are determined by independent approaches and datasets, while SDSL is defined as the residual between observed RMSL, GRD, and VLM. This is adequate given that we do not aim to solve the RMSL budget in a classical sense (i.e., showing that the sum of independently measured individual components matches the total), but rather to decompose HTF into its individual components. We also note that the residuals used here are in good agreement with SDSL estimates inferred from independent temperature and salinity profiles^24^ (Supplementary Fig. 6). Uncertainties are considered by producing 10,000 random samples at each site that account for the errors in the individual components (see Methods). The approach is, again, illustrated for the tide gauge record at Sewell’s Point in Norfolk, Virginia (Fig. 1c, d). Here, VLM shows a linear increase of 3.4 mm per year, while GRD contributions accelerate over the observational period. SDSL is the only component that shows large variability across all temporal scales, including a significant long-term trend. Importantly, when VLM is isolated as the only contributor to RMSL changes, the linear VLM trend does not translate into linear increases in HTF days (Fig. 1d), as the relationship between threshold exceedances and RMSL changes is nonlinear (Supplementary Fig. 7). The changes in the HTF days resulting from isolating the contribution of linear VLM therefore largely reflect tidal and non-tidal residual (surge) variability.

**Fig. 6:**
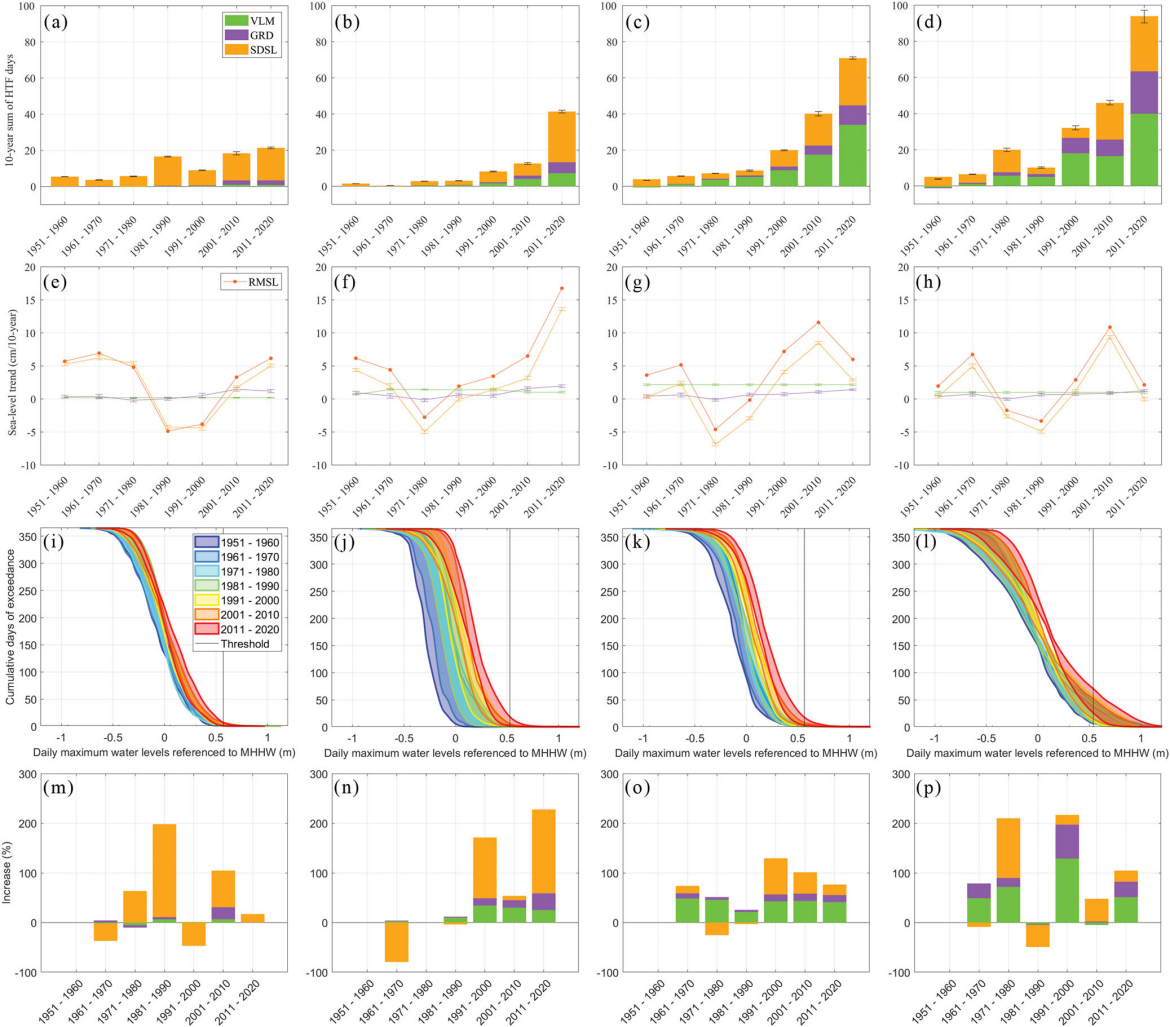
Decadal RMSL trends, HTF days, and their components. The progress of the decadal sum of HTF days, the RMSL trends, and their components are shown for four virtual stations along the CONUS west coasts (**a**, **e**, **i**, **m**; the region A in Fig. 2a), the Gulf of Mexico and south of Cape Hatteras (**b**, **f**, **j**, **n**; the region B in Fig. 2a), the Mid-Atlantic Bight (**c**, **g**, **k**, **o**; the region C up to Woods Hole, Massachusetts in Fig. 2a), and the Gulf of Maine (**d**, **h**, **l**, **p**; the region C north of Woods Hole, Massachusetts in Fig. 2a). The decadal sums of decomposed HTF days for the virtual stations with an error bar showing one standard deviation of all stations (**a**–**d**); the decadal RMSL trends and their contributors are identified by colors in **a** and shown in **e**–**h**; the progress of daily maximum water levels and cumulative annual HTF days are shown for each decade in each region (**i**–**l**). Finally, the percentage increases of decomposed HTF days (compared to the previous decade) are represented in **m**–**p**.

The individual contribution of each component to the changes in HTF days between the control period and the recent 19 years is shown in Fig. 5 (percentage contributions can be found in Supplementary Fig. 3). Along the Atlantic coasts north of Cape Hatteras (Fig. 5c, Supplementary Fig. 3c), VLM and SDSL jointly dominate the increases of HTF days. At sites between Virginia (Sewell’s Point) and New York (The Battery), VLM is the main contributor to the growing HTF, with 2.4–3.6 days per year, accounting for 46–57% of the increases. Farther north, close to the mouth of Long Island Sound (i.e., Woods Hole, Newport, New London, and Montauk), VLM is still the leading process with even higher percentage contributions (48–63%), but the magnitude of the VLM-induced increases in HTF days is less than 1.7 days per year. This is related to the fact that VLM trends decrease northward with increasing distance to the peripheral forebulge center in Southern Virginia/North Carolina showing maximum Glacial Isostatic Adjustment related subsidence^23^ (Supplementary Fig. 8). Sites along the coasts in the Gulf of Maine (Eastport, Bar Harbor, Portland, and Boston) are distinct due to their large increases in HTF days, which may partially be explained by their different local water level characteristics. Interestingly, the GRD component has large percentage contributions (23–32%), with HTF increases of 1.6–3.9 days per year, although the GRD-induced RMSL rise (0.6 mm per year) is not the largest compared to other sites. This is mainly due to further reduction in VLM contributions (24–48%) and large water-level variances (already in the phase where the HTF days increase rapidly with RMSL rise, Fig. 6l) in the Gulf of Maine. At sites that are farther upstream of estuaries or tidal rivers (e.g., Baltimore, Washington D.C., and Providence), SDSL is the leading process to more frequent HTF with 1.5–2.6 days per year (36–56% of the increases). The combination of local factors such as river discharge, tides, and the local wind could result in increased contributions of SDSL to HTF.

South of Cape Hatteras and in the Gulf of Mexico, the SDSL component is often the largest contributor to the increases in HTF days (Fig. 5b). Along the South Atlantic Bight coast, where RMSL changes are largely affected by local^31^ and remote^25,32^ atmospheric and oceanic forcing, the SDSL contributes 1.5–2.0 additional HTF days per year (50–80% of total increase in HTF days) from the control period (Supplementary Fig. 3b). In the Gulf of Mexico, SDSL is still the leading process in increasing HTF occurrences, except for Galveston at Pier 21 and Pleasure Pier, where VLM (47%) and SDSL (45%) are equally important (Supplementary Fig. 3b) and where HTF days have increased faster than elsewhere along the Gulf coast. For the sites on the coast of Texas, nonlinear VLM data is used (see Methods). Compared with the growth of HTF days based on linear VLM, we do not notice significant changes in the contributions of VLM in HTF days between two snap-shot periods, but it affects VLM contributions at decadal to multi-decadal time scales (not shown).

Along the west coasts (Fig. 5a), SDSL is the leading process for HTF changes, but the increases in HTF occurrences are much slower (0.3–3.6 days per year) than in other regions. VLM varies along the west coast. In Friday Harbor, Port San Luis, and Los Angeles, VLM-induced HTF days decreased due to uplift but increased at other sites due to subsidence. Compared to the other two CONUS regions, the sites along the west coast have been less affected by HTF mostly due to slower rates of RMSL rise (1–2 mm per year) caused by dynamical suppression of SDSL rates related to climate-internal decadal variability in the Pacific between the 1980s and early 2010s^33–35^ and small water-level variances (still outside the phase where the HTF days increase more rapidly with RMSL rise, Fig. 6i). However, since 2011 the low-frequency mode of sea-level variability in this region (linked to the Pacific Decadal Oscillation) has undergone a shift toward its positive phase^36^. With ongoing RMSL rise, this will therefore eventually lead to an increase in the frequency of HTF events in the near future.

Along the coasts of some Pacific islands (Fig. 5d), no HTF is detected based on the NOS flooding thresholds at most sites, and only a few sites show less than one day of increase in HTF. In contrast, up to 28 days of increases in HTF (Supplementary Fig. 2) are detected in this region with NWS flooding thresholds (Supplementary Fig. 1). These differences stem from selecting either more broad-vulnerability-focused or more local-feature-focused thresholds^7^. But regardless of the absolute increases in HTF days detected with different flooding thresholds, the percentage contributions of processes change very little in other CONUS regions (see Supplementary Fig. 3, Supplementary Fig. 4). Therefore, we can infer that the SDSL is the dominant process leading the increases in HTF occurrence on the coasts of Pacific islands, while the contributions of VLM highly vary between locations with a range of −21% to 27% (Supplementary Fig. 4d).

### Decadal changes in HTF and drivers

As shown in the example of Sewell’s Point in Norfolk, Virginia, the annual HTF days increase at a nonlinear rate (Fig. 1b) with varying contributions from VLM, GRD, and SDSL over time (Fig. 1d). We therefore also calculate changes in decadal sums of HTF and individual contributors since 1950 for four “virtual stations” that represent the average conditions along the CONUS west coast, the Gulf of Mexico and the South Atlantic Bight, the Mid-Atlantic Bight, and the Gulf of Maine (see Methods, Fig. 6). Again, the largest increases in the HTF over the last seven decades occurred in the Mid-Atlantic Bight and the Gulf of Maine (Fig. 6c, d). Decadal SDSL trends dominate the RMSL budget in these regions with the highest rates (in excess of 10 cm/decade) in the early 2000s (Fig. 6g, h). This also resulted in increases of HTF days of 45–100% compared to the preceding decade (Fig. 6o, p). But in the 2010s, when RMSL rates returned to more moderate (but still increasing) trends, the HTF days increased even faster. This has again to do with the water level distribution at the threshold locations (Fig. 6i–l). Depending on where the threshold is located, even very little changes in RMSL can lead to substantial changes in HTF. Along the CONUS west coast, where the flooding thresholds have still been barely exceeded and RMSL rates were suppressed during the 1980s and 1990s^33^, the HTF was marked by decadal variations dominated by changes in SDSL (Fig. 6a, e, i). More remarkable are the changes in the decadal sums of HTF days in the Gulf of Mexico (Fig. 6b). While the decadal sums were generally even smaller than along the CONUS west coast in the first six decades, HTF days increased by over 220% since 2011 (from 13 to 42 days per decade) (Fig. 6n). This rapid uptake is related to an acceleration in RMSL (rates in excess of 15 cm per decade) and predominantly driven by gyre-scale changes in SDSL^25,37^ (Fig. 6f). In combination with the large rates in nonlinear VLM, the SDSL acceleration has shifted the water level distributions more than in any other regions (Fig. 6j). As a result, even more moderate rise in RMSL will drive faster increases in HTF days in the Gulf region in the coming decades, with potentially severe impacts for the vulnerable coastline and its communities.

## Discussion

Our results significantly advance our understanding in the physical causes of HTF along the coasts of the CONUS and Pacific islands. More than 84% of increases in HTF days can be explained by RMSL rise at most sites, a number that is higher than previous estimates^38^. However, our results indicate that in addition to RMSL, the variance of water levels in combination with the location of the flooding threshold in the water level distribution are important pacemakers of changes in HTF locally. We also demonstrate that the most important drivers of HTF changes vary along the coastline with VLM being the dominant contributor in the northeast and SDSL being most relevant elsewhere and at shorter timescales up to a couple of decades. GRD fingerprints have been becoming increasingly important recently at all locations but still contribute less than VLM and SDSL to the observed increases in HTF days (Fig. 6a–d).

The increase of VLM-related HTF in the northeast is primarily controlled by Glacial Isostatic Adjustment^23^, a natural process that will persist into the foreseeable future (Supplementary Fig. 8). SDSL, however, is influenced by anthropogenic greenhouse gas emissions and is, therefore, at least in the long term, sensitive to greenhouse gas mitigation efforts. This means that communities in the northeast need to adapt to increasing HTF even if climate-related contributions stabilize in the coming decades (e.g., due to enhanced climate mitigation efforts at a global scale). Our results also demonstrate that changes in the frequency of HTF at seasonal to decadal timescales react most sensitive to variations in SDSL, while VLM and GRD components influence HTF more gradually over multi-decadal timescales. Thus, internal climate variability plays a crucial role in modulating periods of enhanced or reduced HTF, and understanding the processes involved in this variability and the relationships to SDSL is critical for accurate predictions of HTF changes along CONUS and Pacific Island coasts. This is illustrated in a sterodynamically driven acceleration of HTF in the Gulf of Mexico over the past decade. More studies are required to clarify the involved oceanographic processes and the drivers of nonlinear VLM to better anticipate and predict the future impacts of HTF.

## Methods

### Tide gauge data and quality controls

We use hourly verified water levels from 41 NOAA tidal gauges along the coasts of the CONUS and Pacific islands. We use only records that provide at least 50% data coverage (counted by hourly data availabilities) over the two comparison periods between 1950–1968 and 2002–2020 (Supplementary Table 1). All hourly water levels are in the metric unit and reference to the contemporary National Tidal Datum Epoch for 1983–2001.

### Calculation of annual HTF days

The HTF days are calculated based on the exceedance of hourly water levels beyond a predefined threshold for minor flooding. In the current study, a HTF day is defined as when any hourly water level in this calendar day exceeds the site-specified minor flooding threshold. The threshold value at each site is either based on ref. ^7^ or calculated using a regression function (Eq. 1) developed by ref. ^4^,

1 $${H}_{{EX}}=1.04* {H}_{{GT}}+0.5$$

where ${H}_{{EX}}$ is the threshold for minor flooding above the gauge’s tidal datum of Mean Higher High Water (MHHW), and ${H}_{{GT}}$ is the local great diurnal tide range in the datums of tabulation of the current NOAA epoch from 1983 to 2001. A HTF day is detected if at least one hourly water level exceeds the threshold (${H}_{{EX}}$). The HTF days are first calculated with hourly verified water levels. To quantify HTF days induced by RMSL changes, two initial steps are required:

1. Define RMSL: We use the MATLAB function *smooth* with a cutoff period of 30 days and a LOWESS filter, so the RMSL contains the seasonal to interannual sea-level variability and also long-term sea-level rise.
2. Define a control period: We define the first 19-year period (1950–1968) as a control period and calculate the RMSL as anomalies relative to that period. We then subtract RMSL from the verified hourly water levels and call the residual control water levels. The control water levels represent the HTF benchmark under 1950–1968 conditions and contain contributions by changes in tides and storminess.

HTF days resulting from RMSL changes are then calculated by subtracting the HTF days based on control water levels from the HTF days detected using the raw hourly verified water levels. HTF days are always expressed in days per year. At locations that have remaining data gaps, it is assumed that the provided coverage is representative for the entire 19-year period (this assumption mainly applies to Rockport and Galveston Pleasure Pier, Texas, where longer data gaps exist; see Supplementary Table 1). We note that the numbers of annual HTF days are sensitive to the choice of the control period, but regardless of the years of the control period, the main conclusion remains that RMSL changes are the main driver behind the increases in HTF occurrence. In the current study specifically, we choose 1950–1968 as the control period. Some sites in southern New England and Puget Sound have their HTF days based on the control water levels slightly reduced from 1950–1968 to 2002–2020, which leads to the contributions of RMSL changes in HTF days exceeding 100% between these two periods. We set an upper limit of 100% in the color scale in Fig. 2a.

### RMSL components

We divide the observed RMSL into three components: barystatic GRD fingerprints, VLM, and SDSL. As GRD fingerprints, we take estimates from ref. ^18^. The estimates consist of a 100-member ensemble in annual time series that takes into account the uncertainties of different observation and reanalysis systems. To use the GRD fingerprints in the contributions analysis of HTF occurrence, annual GRD is interpolated into hourly values by using spline interpolation^39^. This assumes that intra-annual variability in GRD fingerprints is minor, which is reasonable for locations far away from the freshwater source.

VLM is estimated following the same approach as in ref. ^24^, which is based on the difference between observed RMSL and the nearest neighbor series from the hybrid sea level reconstruction^19^. As the hybrid reconstruction estimates sea level from a set of pre-described processes, the differences to a local tide gauge can, similar to the difference between a tide gauge and satellite altimetry, be interpreted as VLM. Reference^24^ demonstrated good correspondence between these estimates and independent estimates from the Global Navigation Satellite System. The advantage of the VLM from the difference between a tide gauge and the hybrid reconstruction is a VLM estimate with monthly time series that goes back in time as far as 1900 (depending on the data availability at individual locations), also allowing for an estimate of nonlinear VLM. Nonlinear VLM has been shown to affect tide gauges along the Texas coastlines^40,41^ significantly, and in former assessments, this nonlinearity has been taken into account by subtracting tide gauge records in Texas from a geologically stable site in the Florida Panhandle region (e.g., Pensacola). Here we estimate nonlinear VLM by applying a Gaussian Process Regression to the differences between the tide gauge records and the hybrid reconstruction. This smoothes the differences and yields very similar results as estimates based on differences to Pensacola. Uncertainties in VLM are estimated assuming that the residuals to the linear trend are normally distributed, temporally correlated, and can be modeled using an autoregressive process of order one. We randomly generate a 100-member ensemble of VLM.

As our goal here is to explain the independent contributions to HTF rather than closing the sea level budget in a classical sense, we interpret the differences between the individual tide gauge records and the sum of GRD and (nonlinear) VLM as SDSL. Thus the SDSL term does not necessarily only contain sterodynamic processes due to steric expansion and ocean circulation changes, but also inverted barometer contributions and any residual process that is not properly represented by the other two components. Uncertainties in the SDSL are estimated by propagating the uncertainties from the two ensembles of GRD and VLM into the residuals. This leads to 10,000 (100 GRD times 100 VLM estimates) SDSL estimates. To validate that the residuals are indeed proper estimates of SDSL in this region, we compare the estimates with a previous study^24^. Reference^24^ estimated SDSL for region averages north and south of Cape Hatteras and for the west coast using temperature and salinity observations in the deep ocean. Our SDSL estimates agree well with the region-wide indices^24^ (Supplementary Fig. 6), which is expressed by strong to very-strong correlation coefficients between r = 0.64 to r = 0.92, underpinning our assumption that the residuals are indeed dominated by SDSL changes.

### Decomposition of annual HTF days

We divide each RMSL-induced HTF day based on the contributions of individual components: GRD, VLM, and SDSL, in the RMSL changes, by assuming that their relative contribution to observed water levels equals their relative contribution to HTF. This is necessary as often a single contributor alone is unable to push water levels over the flooding threshold. Instead, the combination of several components is, in most cases, necessary to produce flooding, but their contributions to the HTF will depend on the different combinations of the components^15^. In this study, we use a method by looking at each hourly exceedance due to RMSL changes and then calculating a water-level fraction of each component in RMSL at these hourly events. For HTF days with multiple hourly exceedances, the fractions of individual components are averaged. With this method, the contributions of components are independent of the order of selections rather than determined by their water-level contribution in the RMSL when a HTF event occurs. The annual HTF days of each component are summarized from its daily fractions. In this way, the summation of the annual HTF days of all components is guaranteed to equal the total annual HTF days resulting from the RMSL changes. This method also naturally assures that the total water levels of components are equal to the RMSL. The annual HTF days presented in this study are the mean of 10,000 member ensemble. Uncertainties are represented as one-sigma standard error (i.e., encompassing 68% of the entire ensemble).

### Regional virtual stations

To reveal the progress of regional decadal RMSL trends, HTF days, and their contributors, one virtual station is built for each region based on the median of stations for each ensemble member. The stations without HTF days or their water-level data that do not fully cover the 2010s are removed. One-sigma standard errors over all ensemble members of the virtual stations for RMSL trends and the sum of HTF days are calculated for each decade.

### HTF days decomposition into GIA and non-GIA VLM

The annual HTF days are calculated similarly to the decomposition of HTF resulting from RMSL changes but by further separating the VLM into GIA and non-GIA contributions. We only use the mean of GRD, SDSL, and VLM in this calculation. For GIA we take the weighted mean from the GIA ensemble^42^.

### Supplementary information

Supplementary

## Data Availability

Processed water-level data, HTF days, and data for re-generating plots are available at 10.5281/zenodo.7677796.
