# Supplementary material for: Causes of accelerated High-Tide Flooding in the U.S. since 1950
Source: NPJ Clim Atmos Sci. 2023 Dec 15;6(1):210. doi: 10.1038/s41612-023-00538-5 (PMC11041669; doi:10.1038/s41612-023-00538-5)
Supplement: Supplementary file 1 — Supplementary [file 41612_2023_538_MOESM1_ESM.pdf]

SUPPLEMENTARY

Sensitivity tests of HTF with NWS minor flooding thresholds

In the main text, we are using the NOS minor flooding thresholds (**Supplementary Fig. 1**) to assess the changes in HTF and contributions of their components. This choice agrees with the NOAA report of *Global and Regional Sea Level Rise Scenarios for the United States*[7] and focuses on the broader vulnerabilities of U.S. coasts to HTF. The NOAA National Weather Service (NWS) also has a system of flooding thresholds to communicate with the public about ongoing or expected coastal flooding hazards. These NWS minor flooding thresholds are often specified with local features and vary more strongly along the coastline (**Supplementary Fig. 1**).

The difference between selected flooding thresholds will essentially affect the detection of HTF events and result in different flooding days, as the HTF, by definition, is the exceedance of observed water levels above a single given height, i.e., the flooding threshold. As excepted, along the coasts of the CONUS and Pacific islands, the increase of HTF days shows different patterns resulting from the NWS minor flooding thresholds (**Supplementary Fig. 2**) compared to those detected with NOW thresholds (**Fig. 2**). Based on the NWS thresholds, the most vulnerable locations to HTF due to RMSL changes are in the South-Atlantic Bight, where the HTF occurs annually for an average of 55 days in Wilmington from the control period. The Mid-Atlantic Bight is another hotspot with a fast-growing number of HTF days, but it shows very heterogeneous spatial patterns. In Sandy Hook, Atlantic City, Lewes, and Washington D.C., we find more than 17 days of HTF increase, but less than seven days are detected in Sewell's Point and Baltimore. The Islands of Hawaii also show a notable growth in HTF from 18 to 28 days, except Nawiliwili where only an increase of two days is found. Although the absolute days of increase in HTF are different, the RMSL rise is still the primary driver for the increase in HTF frequencies at most sites of the CONUS and Pacific Islands (32 out of 37 gauges with more than 84% increases in HTF days induced by RMSL changes).

The decomposed HTF days also change with different choices of flooding thresholds. However, the percentage contributions of individual processes to HTF changes are similar using NWS thresholds (**Supplementary Fig. 4**) or NOS thresholds (**Supplementary Fig. 3**). Although the percentages contributions of these components are similar, the results are not identical because the components' water-level fractions vary in the HTF events.

Linear trends of annual RMSL and the 95th percentile of tide and non-tidal residual

We calculate the linear trends of annual RMSL and 95th percentile of tides and non-tidal residuals to reveal the importance of RMSL in the coastal sea-level rise compared with the contributions from changes in tides and non-tidal residuals (including surges) (**Supplementary Fig. 5a**). We note that only very few of the sites assessed in this manuscript are notably affected by changes in tides (primarily Wilmington, and Gulf of Maine), where the trends in RMSL is comparable to the trends in the annual 95th percentile of tides and residuals. Even in Wilmington, the trends in high percentiles of the tides are smaller than those seen in the RMSL. Additionally, both changes in tides and non-tidal residuals have little effect on the increase in the frequency of HTF (**Supplementary Fig. 5b**).

Supplementary Figures

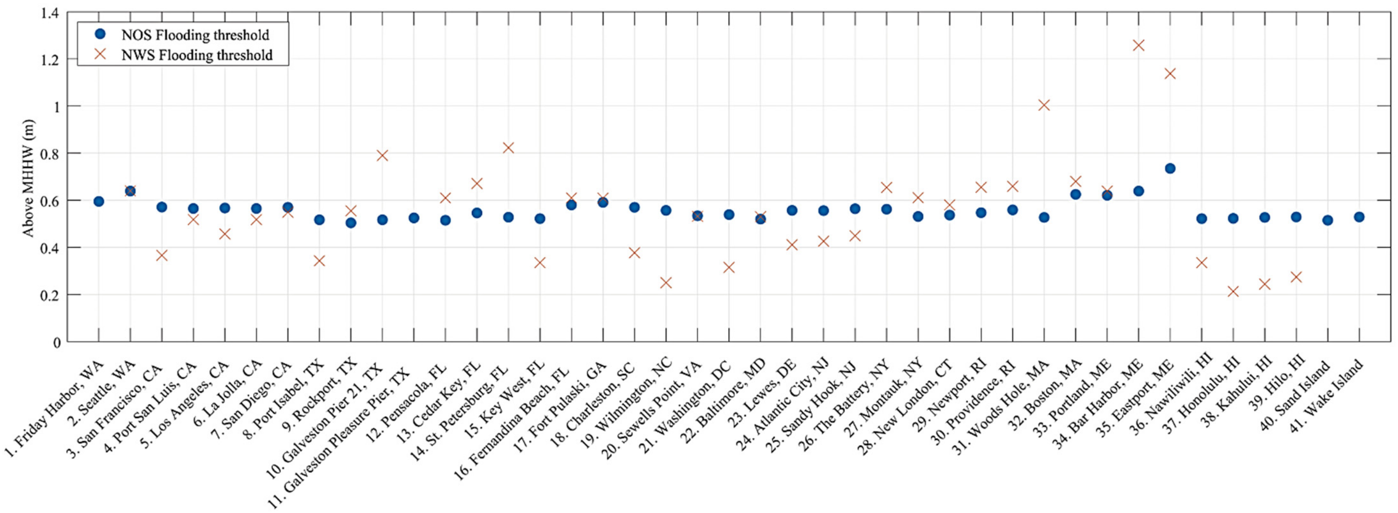

Supplementary Fig. 1 | NOAA minor flooding thresholds. The minor flooding thresholds used by NOAA National Ocean Service (NOS) and National Weather Service (NWS) for all selected sites. The missing values indicate the NWS thresholds are unavailable.

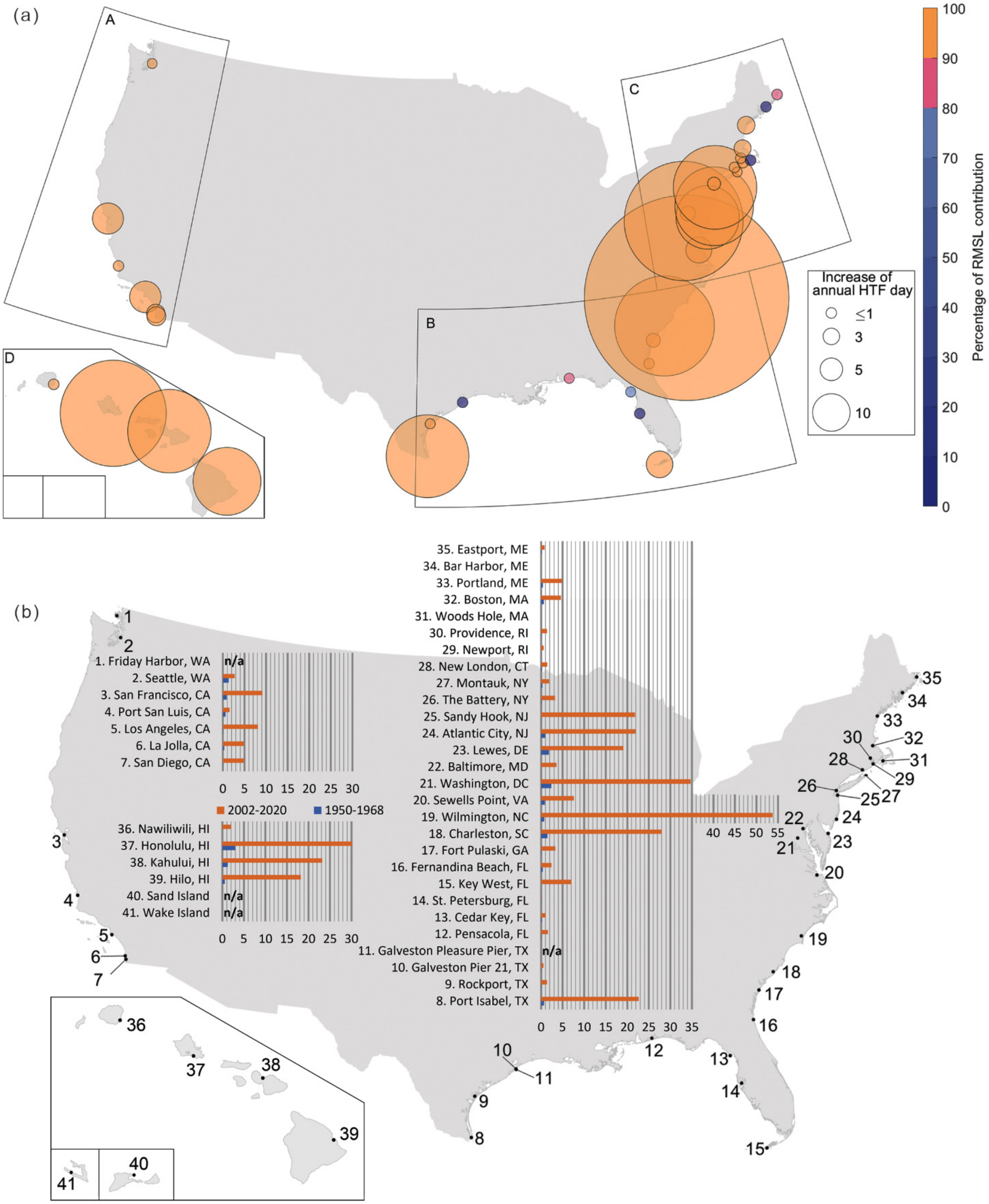

Supplementary Fig. 2 | Increase of annual HTF days and RMSL contributions detected with NWS flooding thresholds. Similar to **Fig. 2** but representing the HTF days detected with the NWS minor flooding thresholds. The sites without NWS flooding thresholds are not shown in (a) and are labeled with n/a in (b).

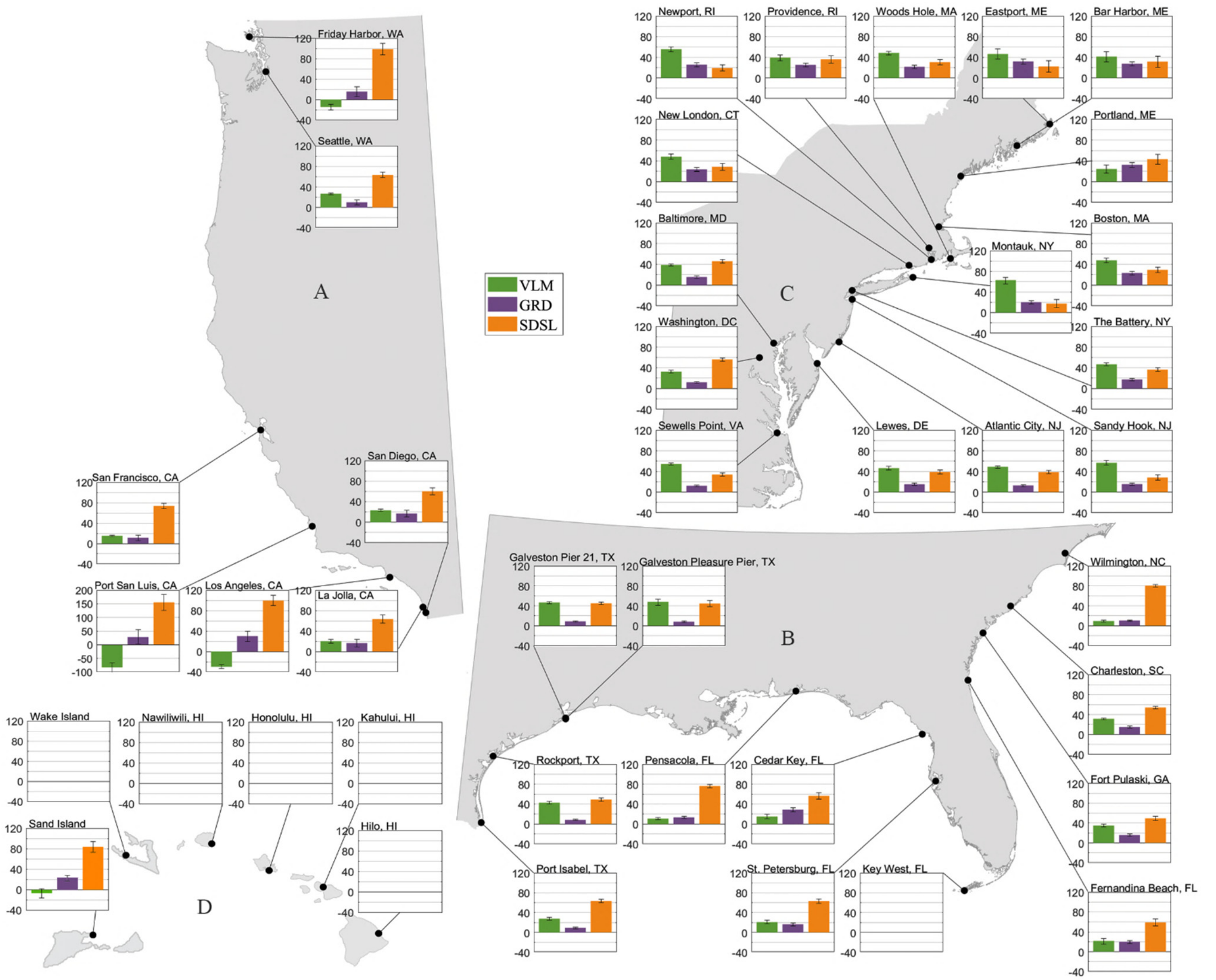

Supplementary Fig. 3 | The percentage contributions of individual processes in the annual HTF detected with NOS flooding thresholds. Similar to **Fig. 5,** but representing the contribution of VLM, GRD, and SDSL in percentages of HTF increases resulting from RMSL changes over two 19-year periods from 1950-1968 to 2002-2020.

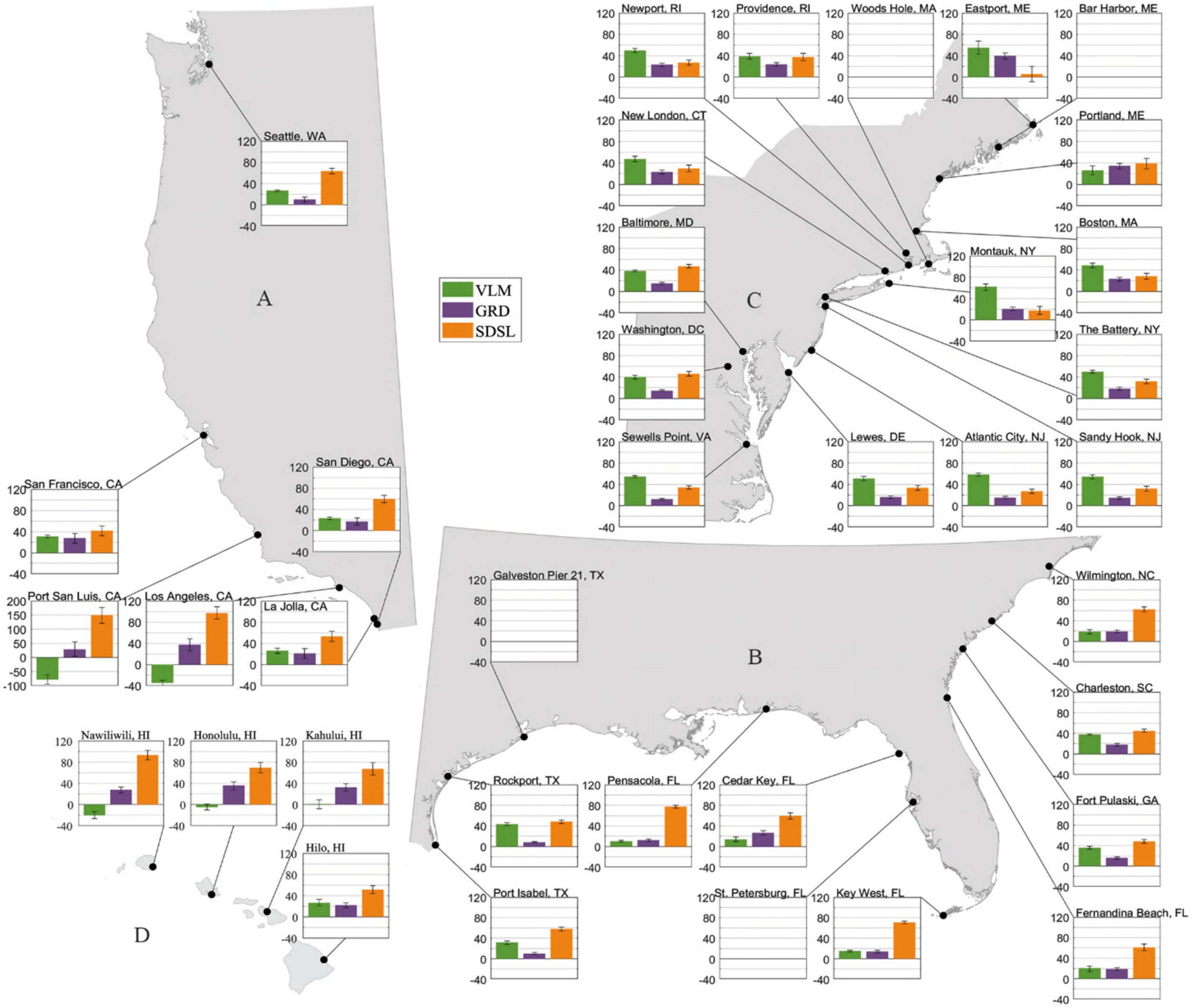

Supplementary Fig. 4 | The percentage contributions of individual processes in the annual HTF detected with NWS flooding thresholds. Similar to **Supplementary Fig. 3** but representing the results detected with the NWS minor flooding thresholds. The sites without NWS flooding thresholds are not shown.

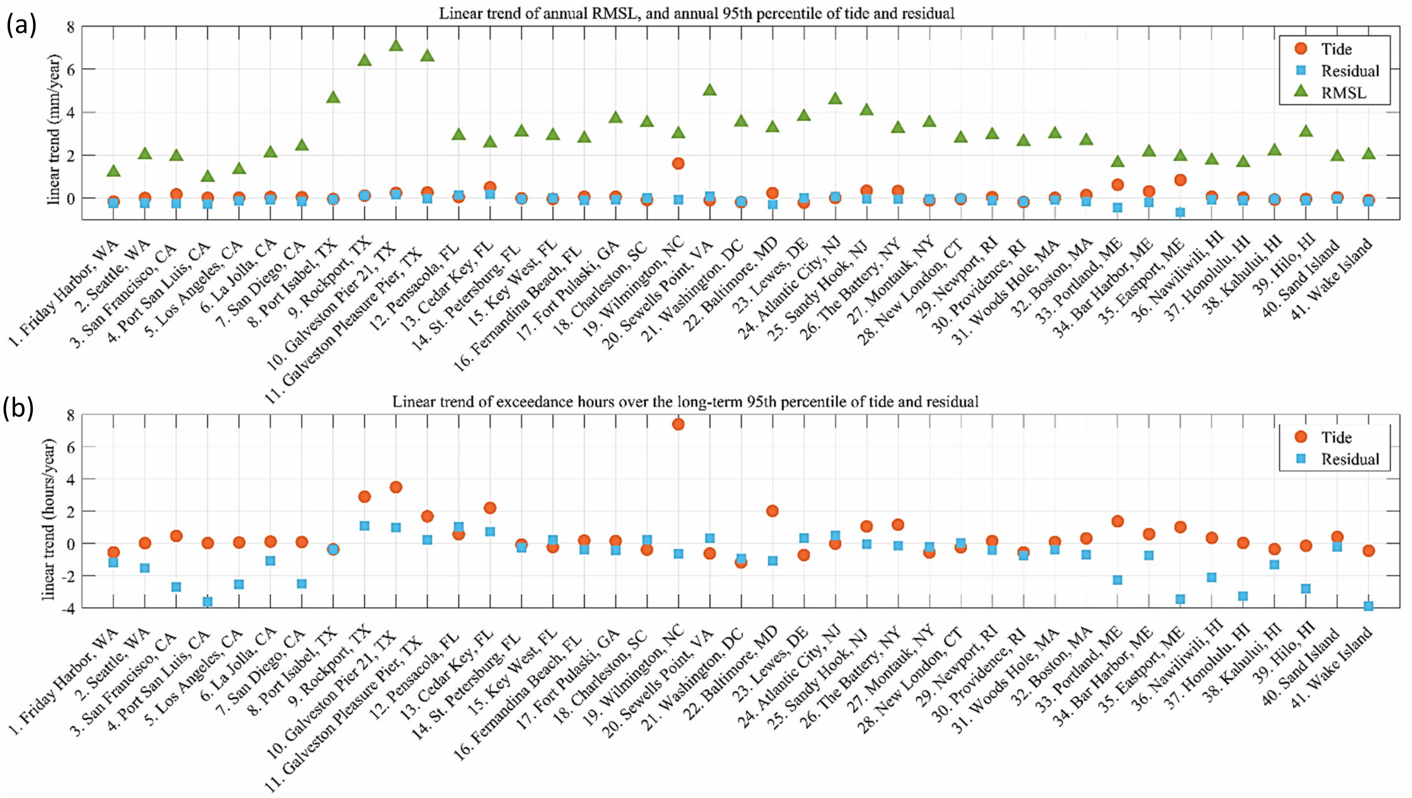

Supplementary Fig. 5 | The trends of RMSL and the annual 95th percentile of tide and non-tidal residual and their exceedance. (a) Linear trends in annual RMSL, the 95th percentile of tides, and non-tidal residuals (including surges) across all sites used in this study, and (b) shows the trends of exceedance hours of the long-term 95th percentile (i.e., change in frequency) in tides and non-tidal residuals at each site. The assessment demonstrates that RMSL is indeed the leading contribution to changes in extreme sea levels. There are only a few isolated examples (Wilmington, Eastport), where changes in tides contribute notably as well.

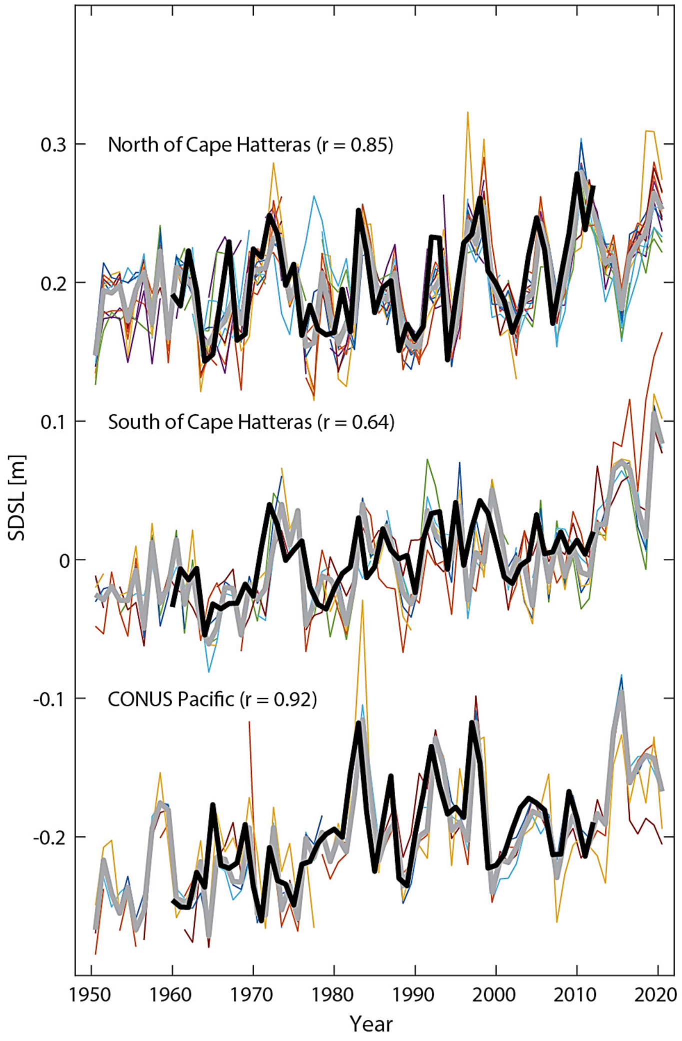

Supplementary Fig. 6 | Validation of residual sea level with SDSL from ref. 24. Shown are the mean residual series (RMSL – VLM – GRD) at each location for the three regions north and south of Cape Hatteras and the CONUS west coast (colored), their region-wide average indices (grey), and the region-wide average indices based on temperature and salinity observations from ref. 24 (thick black). In all regions, the location-specific residual records show good agreement with SDSL reconstructions from ref. 24, which is expressed by correlation coefficients r between the corresponding indices. All time series are based on annual averages.

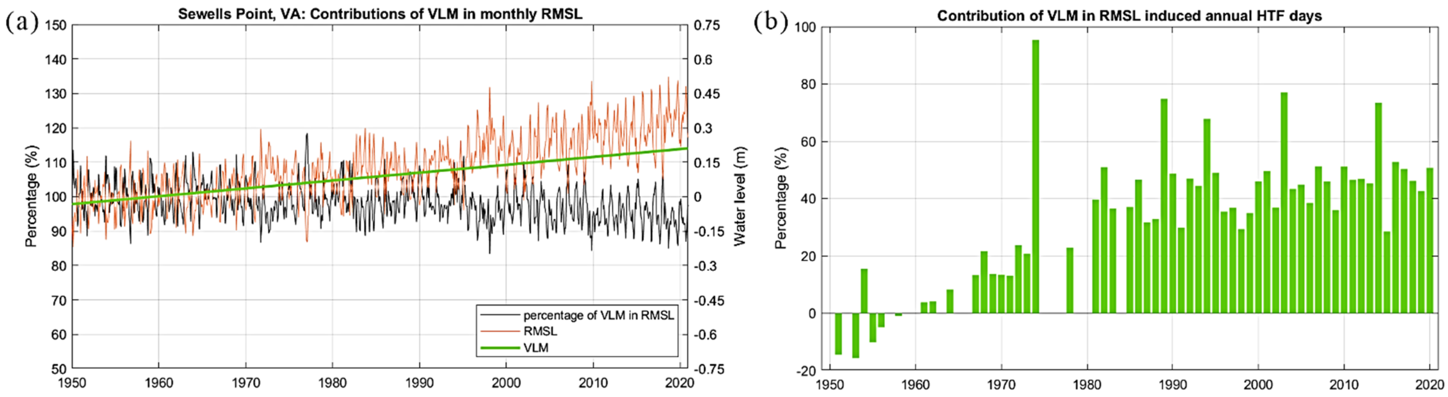

Supplementary Fig. 7 | The percentage contributions of linear VLM in annual HTF days at Sewell's Point in Norfolk, Virginia. (a) The monthly RMSL, the linear VLM, and its time-varying contribution to RMSL in percentage. (b) The time-varying percentage contribution of VLM in annual HTF days.

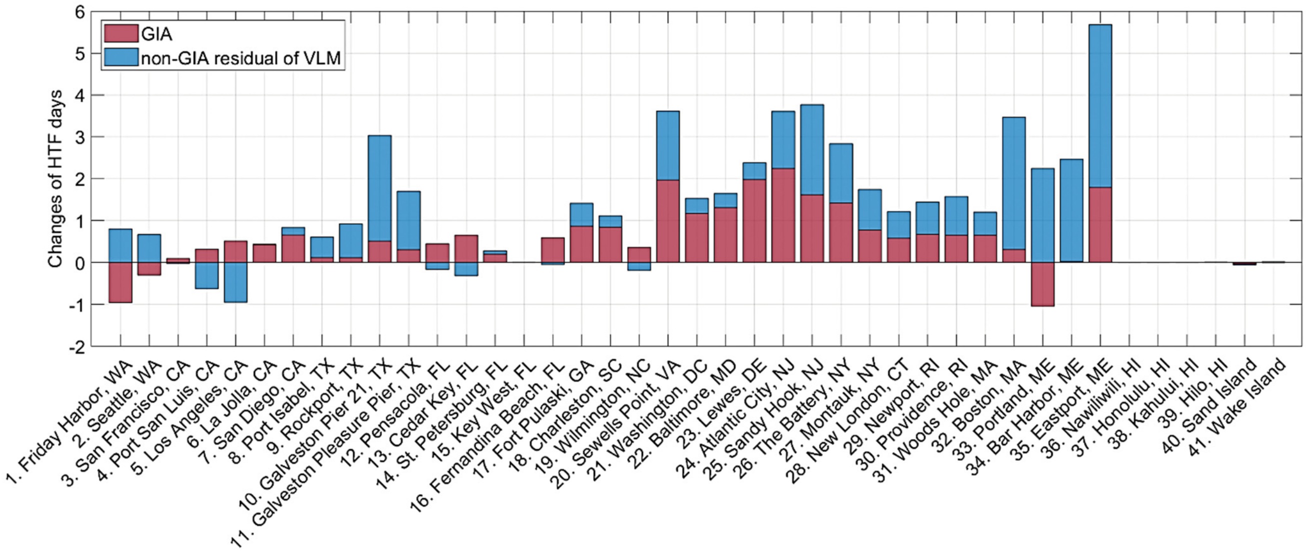

Supplementary Fig. 8 | The annual HTF days changes due to GIA and non-GIA residual of VLM. The changes in HTF days due to the natural GIA and anthropogenic VLM over two 19-year periods from 1950-1968 to 2002-2020.

Supplementary Table. 1 | The data coverage and increase of HTF days in the two 19-year periods.

Name	19-year data coverage		Increase of averaged annual HTF days from 1950-1968 to 2002-2020		
	1950-1968	2002-2020	Total	control water level	RMSL-induced
1. Friday Harbor, WA	92%	100%	0.8	-0.3	1.1
2. Seattle, WA	100%	100%	1.3	-0.1	1.4
3. San Francisco, CA	100%	100%	0.5	0.0	0.5
4. Port San Luis, CA	86%	100%	0.3	-0.1	0.4
5. Los Angeles, CA	99%	100%	1.5	0.1	1.4
6. La Jolla, CA	92%	100%	2.1	-0.1	2.2
7. San Diego, CA	95%	100%	3.6	0.0	3.6
8. Port Isabel, TX	98%	100%	2.2	0.0	2.2
9. Rockport, TX	54%	96%	2.1	-0.1	2.2
10. Galveston Pier 21, TX	100%	100%	6.5	0.0	6.5
11. Galveston Pleasure Pier, TX	55%	50%	3.6	0.0	3.6
12. Pensacola, FL	96%	96%	3.1	0.5	2.6
13. Cedar Key, FL	99%	98%	2.7	0.4	2.3
14. St. Petersburg, FL	93%	100%	1.3	-0.1	1.4
15. Key West, FL	96%	99%	0.1	0.1	0.0
16. Fernandina Beach, FL	96%	99%	2.5	-0.1	2.6
17. Fort Pulaski, GA	99%	100%	4.2	0.1	4.1
18. Charleston, SC	98%	100%	3.6	0.1	3.5
19. Wilmington, NC	96%	100%	1.9	0.0	1.9
20. Sewells Point, VA	99%	100%	6.8	0.1	6.7
21. Washington, DC	100%	94%	4.6	-0.1	4.7
22. Baltimore, MD	98%	100%	4.0	-0.3	4.3
23. Lewes, DE	84%	100%	5.2	0.0	5.2
24. Atlantic City, NJ	98%	98%	7.6	0.2	7.4
25. Sandy Hook, NJ	98%	99%	6.9	0.2	6.7
26. The Battery, NY	99%	98%	6.2	0.1	6.1
27. Montauk, NY	93%	95%	2.8	0.0	2.8
28. New London, CT	95%	99%	2.1	-0.4	2.5
29. Newport, RI	94%	100%	2.3	-0.3	2.6
30. Providence, RI	61%	99%	3.4	-0.6	4.0
31. Woods Hole, MA	81%	98%	2.0	-0.5	2.5
32. Boston, MA	100%	100%	7.5	0.2	7.3
33. Portland, ME	96%	100%	5.2	0.3	4.9
34. Bar Harbor, ME	96%	99%	6.5	0.5	6.0
35. Eastport, ME	90%	99%	13.4	1.1	12.3
36. Nawiliwili, HI	74%	99%	0.0	0.0	0.0
37. Honolulu, HI	100%	100%	0.0	0.0	0.0
38. Kahului, HI	75%	100%	-0.1	-0.1	0.0
39. Hilo, HI	99%	99%	0.0	-0.1	0.1
40. Sand Island	73%	98%	0.8	-0.1	0.9
41. Wake Island	90%	97%	0.2	0.0	0.2
